# Progress in research and development of preventive vaccines for children in China

**DOI:** 10.3389/fped.2024.1414177

**Published:** 2024-07-03

**Authors:** XuYang Zheng, Ge Jin

**Affiliations:** Production Management Department, Beijing Institute of Biological Products Co., Ltd., Beijing, China

**Keywords:** infants, children, vaccination, RV vaccine, HFMD vaccine, PCV

## Abstract

The infant and child stage is an important stage for the continuation and development of human society. The initial years of life have a lasting impact on a child's future. Children under the age of 5 have an immature immune system, especially infants and young children under 6 months of age. At this stage, the population has a low immunity to pathogen infections, making them vulnerable to bacteria and viruses. Vaccination can enhance the immunity of infants and children to specific diseases, reduce the transmission rate of infectious diseases, and promote the development of global public health. This article summarizes the current application status of Rotavirus (RV) vaccine, Hand-foot -mouth disease (HFMD) vaccine, and Pneumococcal Conjugate Vaccine (PCV) in China, as well as the research progress of clinical trial vaccine, laying a foundation for subsequent vaccine development.

## 1 Introduction

In the current world, infants and children are the future of society, and their health status directly affects the development and stability of a country. Therefore, preventing children's diseases and ensuring their healthy growth is the common responsibility and mission of the whole society. Vaccination plays a crucial role in preventing potentially life-threatening infectious diseases and has been proven to be the most cost-effective method (1). Prevention is far more cost-effective than treatment, and timely vaccination can reduce the consumption of medical resources and economic losses caused by diseases. After large-scale vaccination of preventive vaccines in the population, infectious diseases can be effectively prevented and controlled, and it helps to form a herd immune barrier (2).

According to research, RV is the most common pathogen causing severe diarrhea in children under 5 years old worldwide. Every year, 400,000–600,000 children die from RV infection. With the promotion of RV vaccine, the number of deaths per year has decreased to around 200,000 (3). In countries that have not introduced RV vaccine, the proportion of RV detected annually in hospitalized patients with acute gastroenteritis is significantly higher than in countries that have already received RV vaccine (4). HFMD also occurs mostly in children under the age of 5 years. The incidence rate and mortality rate of HFMD have long ranked first among Class C infectious diseases in China, seriously endangering the life and health of children (5). In addition, as one of the important pathogens in humans, *Streptococcus pneumoniae* (*S. pneumoniae*) can cause invasive pneumococcal disease (IPD) (6), especially in children under the age of 5 years, IPD has caused a heavy disease burden globally, including in China (7). In China, most vaccines for children under 5 years old are included in the national immunization program. Through the vaccination of these vaccines, the occurrence of infectious diseases has been effectively controlled. However, vaccines against the three infectious diseases mentioned above are not included in the national immunization program. These non-immunization vaccines can prevent diseases with high incidence and mortality rates, which are equally important in protecting children's health as the national immunization program vaccines. Therefore, this article reviews and summarizes the main vaccines for preventing these three types of infectious diseases that have been listed in China.

## 2 Relevant sections

### 2.1 RV vaccine

RV is a double stranded ribonucleic acid virus that can infect intestinal epithelial cells of infants and young children through fecal pathways, causing cell damage and leading to diarrhea (8). In severe cases, it can cause dehydration and death in infants and young children. The genome of RV mainly encodes 6 non-structural proteins (NSP1-NSP6) and 6 structural proteins (VP1, VP2, VP3, VP4, VP6, VP7) (9). VP6 is the most abundant structural protein and the main determinant of histone reactions (9). The shell proteins VP4 and VP7 determine serotype specificity, forming the basis for the binary classification of viruses (G-type and P-type) (10). According to the RV classification working group survey, 42 G-type and 58 P-type have been isolated globally (11). Due to the independent variation of G-type and P-type, a dual naming system is usually used to determine the strain type (12). Among them, VP7 (G1-G4, G9, and G12) binds to VP4 (P (4), P (6), and P (8), causing most humans worldwide to be infected with RV and leading to diarrhea (13). In recent years, the main serotypes of RV prevalent in China include G1, G2, G3, G4, and G9 (14).

The RV vaccine is the most effective and economical medical means to prevent RV infection. At present, the seven RV vaccines used on the global market are all oral attenuated live vaccines, mainly divided into monovalent vaccines and multivalent vaccines. There are four types of monovalent vaccine, a monovalent attenuated live vaccine (Rotarix) produced by GlaxoSmithKline (GSK) in the UK, a liquid frozen monovalent RV vaccine (Rotavac) produced by Bharat Biotechnology in India, and a monovalent attenuated live vaccine (Rotavin-M1) produced by the Vietnam Biological Products Production and Research Center, a monovalent RV vaccine (LLR) produced by Lanzhou Institute of Biological Products Co., Ltd. There are three types of multivalent vaccine, a pentavalent oral RV vaccine (RotaTeq) produced by Merck, a pentavalent RV vaccine (Rotasiil) produced by the Indian Serum Research Institute, a trivalent attenuated live RV vaccine (LLR3) produced by Lanzhou Institute of Biological Products Co., Ltd.

There are currently three types of RV vaccines on the market in China (as shown in Table 1), namely LLR, LLR3, and RotaTeq. LLR vaccine originated from an RV strain (G10P) isolated from lambs in 1985 (15). After being cultured in calf kidney cells, this strain became the first attenuated RV live vaccine in China to obtain production permits. Children should be vaccinated according to the following immunization program: Children aged 2 months to 3 years old should receive one dose per year (16). The investigation and research after the launch of LLR showed that the LLR vaccine had a protection rate of 35.0%–73.3% against RV gastroenteritis (RVGE), and had higher protection against moderate RVGE caused by G3 serotype (17, 18). After many clinical observations, LLR vaccine is safe and effective, and can significantly reduce the incidence rate of RVGE (19).

**Table 1:** RV vaccine, EV-A71 and PCV13 in China.

Vaccine	Manufacturer	Serotype	Carrier protein	Production cells	Vaccination times
LLR	Lanzhou Institute of Biological Products Co., Ltd.	G10P	/	/	3
LLR3	Lanzhou Institute of Biological Products Co., Ltd.	G2, G3, G4	/	/	3
RotaTeq	Merck	G1, G2, G3, G4 P1A[8]	/	/	3
EV-A71	Institute of Medical Biology of the Chinese Academy of Medical Sciences	/	/	2BS cell	2
EV-A71	Wuhan Institute of Biological Products Co., Ltd.	/	/	Vero cell	2
EV-A71	Sinovac Biotech Co., Ltd.	/	/	Vero cell	2
Prevnar 13	Pfizer	1, 3, 4, 5, 6A, 6B, 7F, 9V, 14, 18C, 19A, 19F, 23F	CRM197	/	4
Weuphoria	Walvax Biotechnology Co., Ltd.	1, 3, 4, 5, 6A, 6B, 7F, 9V, 14, 18C, 19A, 19F, 23F	TT	/	4
Weiminfeibao	Beijing Minhai Biological Technology Co., Ltd.	1, 3, 4, 5, 6A, 6B, 7F, 9V, 14, 18C, 19A, 19F, 23F	TT、DT	/	4

Part of the content was not covered in the manuscript, so it is represented by “/”.

LLR3 is an oral trivalent RV vaccine that recombines with G2, G3, and G4 based on LLR to obtain a trivalent human-sheep RV recombinant strain (20). A phase III clinical study was conducted in Henan, China, involving 9,998 healthy infants aged 6–13 weeks. The research results indicated that the vaccine had good immunogenicity, efficacy, and safety, and had good cross-protective effects. Although it only covers the G2, G3, and G4 serotypes, it can still effectively prevent diarrhea in infants and young children caused by the G1 and G9 serotypes (20).

RotaTeq is a pentavalent oral RV vaccine produced by Merck and is currently one of the most widely used RV vaccines in the world (21). RotaTeq is an oral attenuated live vaccine containing five human bovine RV reassortments [G1, G2, G3, G4, P1A (8)], which can effectively prevent RVGE in infants and young children caused by G1-G4 and G9 (22). This vaccine can be used for vaccination of infants and young children aged 1.5–8 months. In clinical trials, the RotaTeq vaccine has been proven to be highly effective (>85%) in reducing severe RV disease in both developed and developing countries (23). However, the efficacy of vaccines in underdeveloped regions such as Africa was lower than that in developed countries (24). But the vaccine can also cause adverse reactions to the gastrointestinal tract, including diarrhea, vomiting, etc., by stimulating the intestinal immune response (25).

The Wuhan Institute of Biological Products has developed a 6-valent gene reassortment vaccine by reassorting six human VP7 genes and bovine genes and has completed the third phase of clinical trials. The vaccine covers six popular serotypes, including G1-G4, G8, and G9. In the Phase I clinical trial, 120 healthy infants aged 6–12 weeks were studied and demonstrated good safety and immunogenicity (26). In the Phase III clinical trial, a multicenter, efficacy, safety, and immunogenicity clinical study was conducted on 6,400 infants aged 6–12 weeks. The results showed that the protection rates of oral hexavalent reconstituted RV live vaccine against gastroenteritis, severe gastroenteritis, and hospitalization due to gastroenteritis caused by the serotype RV contained in the vaccine were 69.21%, 91.36%, and 89.21%, respectively. This study showed that oral hexavalent reconstituted RV live vaccine has good protective effects on Chinese infants (14).

### 2.2 HFMD vaccine [enterovirus A71 (EV-A71) vaccine]

HFMD is a common childhood disease caused by various enteroviruses, often occurring in children under the age of 5 (27). Among them, coxsackie virus A16 (CVA16) and EV-A71 are the most common, with approximately 90% of HFMD caused by EV-A71 (28). After being infected with these viruses, rash will grow on the mouth, hands, and feet, and the distribution characteristics are obvious. Mild symptoms usually recover well about a week after symptom treatment. However, when the symptoms of the disease are severe, conditions such as encephalitis and pulmonary edema may occur, which are life-threatening (5) and cause a significant financial burden to the patient's family (29, 30).

In response to HFMD caused by EV-A71, the Institute of Medical Biology of the Chinese Academy of Medical Sciences has taken the lead in developing the inactivated EV-A71 vaccine (2BS cell, as shown in Table 1), The vaccine was administered to children aged 6 months to 3 years old. The protection rate against HFMD caused by EV-A71 can reach 97.3%. The research findings were published in the world's top medical journal (the New England Journal of Medicine) in 2014 (31). In addition, in Phase IV clinical trial, the vaccine showed an overall protective efficiency of 89.7% against EV-A71 and an incidence of adverse events of 4.58% through long-term observation of vaccinated children for up to 14 months (32). Through the Phase IV clinical trial, the vaccination target of this vaccine has been changed from children aged 6 months to 3 years old to children aged 6 months to 5 years old. The vaccine has effectively reduced the incidence rate of HFMD in children in China, reduced severe cases and deaths, and protected children's lives and health. The EV-A71 inactivated vaccine (Vero cell, as shown in Table 1) developed by Wuhan Institute of Biological Products Co., Ltd. was launched in 2017. This vaccine is suitable for children aged 6 months to 3 years old. In June 2013, THE LANCET published the first clinical study on the efficacy evaluation of inactivated EV-A71 vaccine in China. This study reported for the first time the effectiveness of the EV-A71 inactivated vaccine in children, covering 22,123 healthy infants and young children aged 6–35 months. Research indicated that the protection rates of EV-A71 vaccine against EV-A71-induced diseases, HFMD, and hospitalized/severe cases of HFMD were 81.85%, 90.93%, and 100%, respectively (33).

The EV-A71 inactivated vaccine (Vero cell, as shown in Table 1) produced by Sinovac Biotech Co., Ltd. was launched in June 2016 to prevent HFMD caused by EV-A71 infection in children aged 6 months to 3 years old. The vaccine showed a protective efficiency of 94.8% against EV-A71-related HFMD in Phase III clinical trials of children aged 6–35 months (34). In order to provide more children with EV-A71 vaccine protection, a phase III clinical trial was conducted in children aged 36–71 months and completed in 2019. The research results showed that after receiving two doses of vaccine, the neutralizing antibody positivity rate of children in this age group was significantly higher than that of the control group in the same age group, demonstrating good safety and immunogenicity (35). In June 2021, the age range of the vaccine was extended to children aged 6–71 months, providing EV71 vaccine protection for a wider range of age groups.

### 2.3 PCV (13-valent PCV, PCV13)

*S. pneumoniae* also known as *pneumococcus,* is a type of *streptococcus,* can cause varying degrees of infection (36). For example, acute otitis media, sinusitis, pneumonia and meningitis are common causes of incidence rate and mortality in the pediatric population (37). *S. pneumoniae* can be classified into more than 90 serotypes based on the specificity of its capsule polysaccharides, with more than 20 serotypes being truly pathogenic (38). There are currently three types of PCV13 listed in China (as shown in Table 1), namely Prevnar 13 vaccine produced by Pfizer, Weuphoria vaccine from Walvax Biotechnology Co., Ltd., and Weiminfeibao vaccine from Beijing Minhai Biotechnology Co., Ltd. These three vaccines cover the same serotypes of *S. pneumoniae*, including 13 serotypes: 1, 3, 4, 5, 6A, 6B, 7F, 9V, 14, 18C, 19A, 19F, and 23F. Among them, 3, 5, 6A, 6B, 9V, 14, 18C, 19A, 19F, and 23F are the most common serotypes in China, with a clear regional distribution (39, 40). From this perspective, PCV13 for children can be used for active immunity in infants and young children to prevent *S. pneumoniae* infection.

Prevnar 13 is the only imported original PCV13 in China, using CRM197 as a protein carrier to overcome the deficiency of polysaccharide vaccine in inducing immune responses in infants and young children under 2 years old. CRM197 protein is a mutant of diphtheria toxin (DT), which loses its cytotoxicity due to the loss of ADP ribosyl transferase activity (41). It can serve as a carrier protein for polysaccharide vaccines, effectively enhancing the immunogenicity of polysaccharide antigens, and is widely used in the field of vaccine development (42). In 2023, the vaccination age for PCV 13 was extended from 6 weeks to 15 months old to 6 weeks to 5 years old in China. Because a large amount of clinical data has proven that the vaccine has high safety and immunity in children aged 6 weeks to 5 years old, and reduces the incidence of IPD (43–45). After the introduction of PVC13, the incidence of invasive pneumonia in 8 children's hospitals in the United States significantly decreased (46). For the pediatric population, PCV13 was initially approved for use in infants and children under 5 years old, as the effectiveness and safety of clinical data has led to the approval of the vaccine for expanded use (6 weeks to 17 years old) in the European Union and the United States (47).

As the first independently developed PVC13 in China, Weuphoria is the second PVC13 globally. The vaccine uses tetanus toxoid (TT) as the viral vector. The carrier protein is an inactivated TT that has been treated with formaldehyde (48). There may be residual formaldehyde and incomplete detoxification of TT during production (49), and close attention should be paid to production testing. The Phase III clinical trial data of Weuphoria showed that the positive rate and geometric average titer of antibodies against 13 serotypes of pneumococci were not inferior to similar imported products. This vaccine has good effects on infants and children aged 6 weeks to 5 years (before their 6th birthday) (50). A recent study showed that there was no significant difference in adverse reactions after vaccination with Weuphoria and Prevnar 13, and most of the adverse reactions were mild and common (48).

Weiminfeibao vaccine uses two carrier proteins (TT/DT) to bind to pneumococcal capsule polysaccharides, making it the world's first dual-carrier PCV13. The single and repeated administration of the vector may affect the immunological efficacy after vaccination, or lead to immune suppression caused by the vector (51, 52). The use of dual carriers can avoid the inhibitory effect of a single carrier protein competing with helper T cells on polysaccharide immune response. The Phase III clinical trial data of Weiminfeibao vaccine (53) showed that the antibody positivity rate and geometric mean titer against 13 serotypes of pneumococci were not inferior to similar imported products, and had good immune effects on infants and children aged 7 months to 5 years.

## 3 Discussion

### 3.1 RV vaccine

In recent years, some progress has been made in the development of RV vaccine. Three types of RV vaccines have been launched in China, as introduced earlier. From a global perspective, there are still four types that have not been listed in China. The monovalent attenuated live vaccine Rotarix produced by GSK is derived from human RV and causes minimal allergic reactions after direct administration. Although it is a monovalent vaccine, clinical studies have shown that it can provide cross-protection against most serotypes of RV (54). The first self-developed monovalent human bovine recombinant vaccine (Rotavac) from India has good immunogenicity against RV with serotype G9P (11, 55). In addition, the freeze-dried pentavalent vaccine produced in India can be stored at 2–8℃ for 30 months. The phase III clinical trial data of Kulkarni et al. showed that the incidence rate of RVGE in the vaccine group was significantly lower than that in the placebo group (56). The Rotavin-M1 vaccine was approved for sale in Vietnam in 2014 and its immunogenicity was investigated in different doses of infants and young children in Vietnam. The results showed that two doses of the Rotavin-M1 vaccine had good immunogenicity in this stage of the population in Vietnam (57). However, the above-mentioned vaccines have not yet been introduced to China, but the effectiveness and safety demonstrated by clinical data are worth our reference, and the development path of vaccine is worth learning from.

With the widespread use of RV vaccines, the prevalence of RV in different regions of the world has also changed, and new dominant strains of RV are constantly changing, posing challenges to the protective effects of the existing RV vaccines. At present, there are potential safety issues with commercialized oral attenuated live vaccines, as well as poor immune efficacy in some low-income countries. It is necessary to develop safer and more efficient multi-valent or multivalent vaccines to prevent the troubles caused by RV in humans.

The RV-attenuated live vaccines increases the risk of intussusception due to oral administration (58). After receiving the RotaTeq vaccine in Mexico, there was 1 case of intussusception per 51,000 infants, and after receiving the RotaTeq vaccine in Australia, there were 5.6 cases of intussusception per 100,000 infants (59). In 2016, a study by Tate, J.E et al. showed a small increase in intussusception hospitalizations in children between 8 and 11 weeks after most first doses of the vaccine, but a significant decrease in RV disease, and the benefits of RV vaccination outweighed the increased risk of intussusception (60). In 2021, A review published by Bergman, H et al. pointed out that Rottarix, RotaTeq, Rotasil, and Rotavac can prevent RV diarrhea attacks without increasing the risk of serious adverse events, including intussusception (61). Previous studies have shown that non-replicative RV vaccines immunized through non gut routes produce good protective antibodies in volunteer vaccine research trials (62). Many vaccines for non-intestinal immunity are currently research hotspots, such as subunit vaccines, VLP vaccines, inactivated vaccines, etc., which avoid the shortcomings of some oral vaccines and improve the effectiveness and accessibility of vaccines. The P2-VP8-P (8) recombinant subunit vaccine, as a candidate vaccine for extraintestinal RV, is formed by fusing the VP8 truncated subunit with the P2 epitope of TT. In 2016, a study evaluated the safety and immunogenicity of different doses of P2-VP8-P (8) subunit vaccine. 528 infants were enrolled in this experiment, and the experimental group's anti-P2-VP8 IgG serum showed significantly higher antigen responses to P (4), P (6), and P (8) compared to the control group (63). At present, there are inactivated RV vaccines (IRVs) based on the G1P (8) strain, which have shown good immune and protective effects in animal experiments. After administration to mice, they produced strong serum antibodies and could also induce intestinal mucosal immunity (64). In addition, RV-VLP vaccines have also developed rapidly. As early as 1987, Estes et al. (65) synthesized the main capsid antigen of RV using a rod-shaped virus expression system. Up to now, RV-VLP has been prepared by co-expressing proteins such as VP2, VP4, VP6, and VP7 using a rod-shaped expression system, and their immunogenicity has been evaluated in mouse experiments (66). A recent study combined Norovirus VLP and recombinant RV VP6 protein produced by the baculovirus insect cell expression system to form a vaccine for the protection of acute gastrointestinal viral diarrhea in children (67). However, the vaccine has only been evaluated for immunogenicity and safety in animal models and has not yet been tested in humans. In addition, studies have shown that compared to subunit vaccines, lower doses of antigens are sufficient to trigger similar immune responses in VLP vaccines (68). With the deepening of research, it is believed that the development technology of RV vaccine will become more mature shortly, and new vaccine will also be safer, more efficient, and more cost-effective.

### 3.2 HFMD vaccine

There are over 20 types of enteroviruses that cause HFMD, including EV-A71, CVA16, CVA10, CVA6, and so on (69). Since the independently developed EV-A71 inactivated vaccine was approved and promoted for use in China in 2015, the positive detection rate of EV-A71 in HFMD cases has significantly decreased compared to before. The conversion of dominant strains of HFMD has been monitored in many places, and CVA6 and CVA10 have gradually become dominant strains (70, 71). The prevalence of HFMD caused by CVA10 infection is generally more prevalent in the eastern and central regions, and relatively less prevalent in the western regions (72–74). CVA10 infection can also lead to severe HFMD, and CVA10 infection is more likely to cause Herpangina (HA) (75). Since July 2018, CVA6 has become the main pathogenic serotype of HFMD, accounting for 63.5% and 36.2% of mild and severe patients, respectively (76). However, there are currently no preventive vaccines or specific therapeutic drugs targeting CVA6 and CVA10. The complex and variable HFMD pathogens pose a serious challenge for better control of the HFMD epidemic. Therefore, accelerating the development of HFMD multivalent vaccines with high efficiency and broad-spectrum protection, including CVA6, CVA10, and CVA16, is an inevitable trend.

Immune interference is a technical challenge in studying multivalent inactivated vaccines compared to monovalent inactivated vaccines (77). In 2018, the bivalent inactivated CVA6/CVA10 whole virus vaccine developed by Zhang et al. (78) showed no immune interference in the immune response and effectively stimulated the body to produce neutralizing antibodies, capable of simultaneously neutralizing CVA6 and CVA10. Subsequently, the trivalent inactivated vaccine (CVA6/CVA16/EV-A71) developed by Caine et al. was observed to provide complete protection against fatal attacks on EV-A71 and CVA16 in mouse models. In addition, serum passive transfer studies targeting trivalent vaccine have demonstrated the importance of neutralizing antibodies against CVA6 in controlling HFMD-related enterovirus infections (79). In addition, the trivalent inactivated vaccine (CVA16, CVA10, and CVA6) developed by LIM et al. can induce humoral immunity to produce neutralizing antibodies with protective effects (77). In 2016, Liu et al. preliminarily evaluated the immunogenicity of the tetravalent inactivated vaccine (CVA6/CVA10/CVA16/EV-A71) in a mouse model (78). The vaccine can induce mice to produce specific neutralizing antibodies against these four immunogens, further demonstrating the feasibility of the HFMD multivalent inactivated vaccine.

Compared to the traditional vaccine development route, virus-like particle(VLP) vaccine maintain the structure of viral capsid proteins under the condition of no genetic material, and can simultaneously induce efficient cellular and humoral immunity, with high immunogenicity and safety (80). The current research and development of HFMD VLP vaccine are progressing rapidly. Various serotypes of monovalent and multivalent VLP vaccines have produced corresponding neutralizing antibodies with protective effects against relative pathogens (81–84). But these vaccines have only shown good immunogenicity in animal trials, and clinical trial data in humans remains to be verified. The EV-A71 VLP vaccine prepared by Wang et al. (83) has been approved for Phase I clinical trials and is expected to become the next HFMD vaccine to be launched.

### 3.3 PCV vaccine

*S. pneumoniae*, as a common pathogen, is a common cause of incidence rate and mortality in the pediatric population. In 2017, in China, due to infection with *S. pneumoniae*. The pandemic has caused 570,000 cases of children under the age of 5 years, with 8,010 deaths among them (85). The continuous use of antibiotics in controlling diseases caused by pneumococci has led to some serotypes having resistance to certain antibiotics reaching over 80%, making treatment difficult (86). The disease caused by *S. pneumoniae* urgently requires vaccine to control it, in order to reduce the damage to the human immune system.

In 2000, Wyeth developed the world's first PCV7 vaccine (4, 6B, 9V, 14, 18C, 19F, and 23F), which was launched in the United States and approved for use in infants and children under 5 years old (87). It was also launched in China in 2008. According to statistics, after PCV7 vaccination, the incidence rate of IPD among infants and young children decreased by more than 80% (88). Wang et al. conducted a study in China on children aged 2–5 who received a single dose of PCV7. The results showed that the average concentration of all 7 serotypes of IgG in the experimental group was significantly higher than that in the control group, and there were no serious adverse reactions. The vaccine has good immunogenicity and safety for this age group (89). The PCV10 (Synflorix) (90) vaccine, produced by GSK, contains serotypes 1, 4, 5, 6B, 7F, 9V, 14, 18C, 19F, and 23F, suitable for infants and young children aged 6 weeks to 2 years. In 2006, a study showed that the vaccine had a similar response rate to the serotype antibodies shared by PCV7, and added three serotypes, which could prevent more IPD (91). In 2010, the PCV13 (Prevnar 13) developed by Pfizer was used in the United States, and in 2016, studies showed that the protective effect on the common serotypes of PCV7 was equivalent, and the incidence rate of the new serotypes 1, 3, 5, 6A, 7F and 19A decreased significantly (92). The PCV15 (Vaxneuvance) vaccine produced by MSD includes serotypes 1, 3, 4, 5, 6A, 6B, 7F, 9V, 14, 18C, 19A, 19F, 23F, 22F, and 33F. With the support of safety and immunogenicity data in different stages of the population, it is suitable for pediatric population from 6 weeks to under 18 years old and adult population from 18 years old and above. Vaxneuvance produced robust immune response to each of the 15 serotypes as assessed by IgG GMCs and response rates, and the vaccine had a similar response rate to the common serotypes of PCV13, with higher response rates for serotypes 22F and 33F (93). The PCV20 (Prevnar20) produced by Pfizer has added 7 serotypes on top of Prevnar13 and is currently suitable for people aged 18 and above. A recent Phase 3 study in Japan, Prevnar20 was injected into healthy infants aged 2–6 months. The results showed good safety and tolerability to healthy infants, and it can induce a strong serotype specific immune response (94). In a phase 3 clinical study conducted in the United States, the results showed that Prevnar20 can trigger a strong serotype specific immune response with good tolerance, which is expected to help protect infants and young children from the impact of pneumococcal disease caused by 20 serotypes (95).

In addition, several 23 valent pneumococcal polysaccharide vaccines (PPV23) listed in China contain 1, 2, 3, 4, 5, 6B, 7F, 8, 9N, 9V, 10A, 11A, 12F, 14, 15B, 17F, 18C, 19F, 19A, 20, 22F, 23F, 33F and other serotypes, which can prevent related serotype *S. pneumoniae* infectious diseases. However, because the capsular polysaccharide is a T-cell-independent antigen, it cannot trigger the protective immune response in infants with an imperfect immune system, and can only be used for children and adults over 2 years old (96).

Some studies show that the incidence rate of IPD in children under 5 years old will decrease significantly after PCV is included in national immunization (97). The World Health Organization recommends that countries include PCV in their child immunization plans, but in China, the PCV vaccine is a non-immunization plan vaccine. The PCV vaccine, as the most effective and economical means of preventing pneumococcal diseases, has a much lower PCV vaccination rate in China than at the international level, and the indirect vaccination rate is uneven in different regions. Only by accelerating the research and development of pneumonia vaccines and reducing production costs can the vaccination rate be improved. Currently, according to Insight data, multiple companies such as CanSino Biologics Inc., Lanzhou Institute of Biological Products Co., Ltd., Sinovac Biotech Co., Ltd., and AIM Vaccine Co., Ltd. are also conducting research and development on the PCV13, and are looking forward to the release of clinical data.

## 4 Conclusions

At present, vaccines administered to infants, young children, and children in China include planned immunization vaccines and non-planned immunization vaccines. For example, BCG vaccine, IPV vaccine, hepatitis B vaccine, etc., the coverage rate of such immunization programs is very high, which greatly eliminates the harm of these infectious diseases to children. However, non-immunization vaccines such as RV vaccine, HFMD vaccine, PCV13 vaccine, etc., due to their high manufacturing costs, have a much lower vaccination rate for this group of people compared to developed countries abroad. Vaccines can only improve health and prevent deaths if they are used, and immunization schedule must be able to achieve and sustain high vaccine uptake rates. Vaccine hesitation is an increasingly important issue for national immunization plans. The emergence of vaccine hesitancy (98) is mainly due to concerns about vaccine safety and disregard for the harm of infectious diseases. We need to timely strengthen the audience's awareness of vaccines and the diseases they prevent, and reduce vaccine hesitancy by increasing the enthusiasm of medical personnel, increasing vaccine supervision, and establishing trust monitoring mechanisms. In addition, it is necessary for companies and countries producing vaccines to lay out and develop safer, more efficient, and more cost-effective new vaccines as soon as possible.

Safety should be the primary consideration before vaccination for infants and young children. The effectiveness of vaccine such as RV vaccine, HFMD vaccine, and PCV13 vaccine listed in China is evident, but there are still different immune reactions among individuals, which may cause mild adverse reactions such as local redness and swelling, fever, and serious adverse reactions such as allergic reactions and neurological complications. Therefore, in clinical trials and after the launch of vaccine, it is necessary to monitor and evaluate adverse reactions after vaccination, promptly identify and address potential safety issues, and ensure the health and safety of vaccinators.

## References

[1] Maciosek MV Coffield AB Edwards NM Flottemesch TJ Goodman MJ Solberg LI. Priorities among effective clinical preventive services: results of a systematic review and analysis. Am J Prev Med. (2006) 31:52–61. 10.1016/j.amepre.2006.03.012 16777543

[2] Smith DR. Herd immunity. Vet Clin North Am Food Anim Pract. (2019) 35:593–604. 10.1016/j.cvfa.2019.07.001 31590904

[3] Tate JE Burton AH Boschi-Pinto C Parashar UD. Global, regional, and national estimates of rotavirus mortality in children <5 years of age, 2000–2013. Clin Infect Dis. (2016) 62(Suppl 2):S96–105. 10.1093/cid/civ1013 27059362 PMC11979873

[4] Aliabadi N Antoni S Mwenda JM Weldegebriel G Biey JNM Cheikh D Global impact of rotavirus vaccine introduction on rotavirus hospitalisations among children under 5 years of age, 2008–16: findings from the global rotavirus surveillance network. Lancet Glob Health. (2019) 7:e893–903. 10.1016/S2214-109X(19)30207-4 31200889 PMC7336990

[5] Saguil A Kane SF Lauters R Mercado MG. Hand-foot-and-mouth disease: rapid evidence review. Am Fam Physician. (2019) 100:408–14. 31573162

[6] Jackowska T Pluta J. Routine infant immunization with the 7- and 13-valent pneumococcal conjugate vaccines: current perspective on reduced-dosage regimens. Arch Med Sci. (2012) 8:542–8. 10.5114/aoms.2012.29535 22852013 PMC3400920

[7] Wang CP Lin YT Du YZ Zhang T Wang YY Wang YJ Impact of innovative immunization strategy on PCV13 vaccination coverage among children under 5 years in Weifang city, China: a retrospective study. Vaccine. (2024) 42:1136–44. 10.1016/j.vaccine.2024.01.030 38267332 PMC10911081

[8] Carvalho MF Gill D. Rotavirus vaccine efficacy: current status and areas for improvement. Hum Vaccin Immunother. (2019) 15:1237–50. 10.1080/21645515.2018.1520583 30215578 PMC6663136

[9] Steele JC. Rotavirus. Clin Lab Med. (1999) 19:691–703. 10.1016/S0272-2712(18)30111-2 10549433

[10] Ward RL Bernstein DI. Rotarix: a rotavirus vaccine for the world. Clin Infect Dis. (2009) 48:222–8. 10.1086/595702 19072246

[11] Rotavirus Classification Working Group: RCWG. (2023). Available online at: https://rega.kuleuven.be/cev/viralmetagenomics/virus-classification/rcwg (Accessed March 31, 2024).

[12] Sadiq A Bostan N Yinda KC Naseem S Sattar S. Rotavirus: genetics, pathogenesis and vaccine advances. Rev Med Virol. (2018) 28:e2003. 10.1002/rmv.2003 30156344

[13] Dóró R László B Martella V Leshem E Gentsch J Parashar U Review of global rotavirus strain prevalence data from six years post vaccine licensure surveillance: is there evidence of strain selection from vaccine pressure? Infect Genet Evol. (2014) 28:446–61. 10.1016/j.meegid.2014.08.017 25224179 PMC7976110

[14] Wu Z Li Q Liu Y Lv H Mo Z Li F Efficacy, safety and immunogenicity of hexavalent rotavirus vaccine in Chinese infants. Virol Sin. (2022) 37:724–30. 10.1016/j.virs.2022.07.011 35926726 PMC9583109

[15] Buchy P Chen J Zhang XH Benninghoff B Lee C Bibera GL. A review of rotavirus vaccine use in Asia and the pacific regions: challenges and future prospects. Expert Rev Vaccines. (2021) 20:1499–514. 10.1080/14760584.2020.1853532 33275065

[16] Wang Y Li J Liu P Zhu F. The performance of licensed rotavirus vaccines and the development of a new generation of rotavirus vaccines: a review. Hum Vaccin Immunother. (2021) 17:880–96. 10.1080/21645515.2020.1801071 32966134 PMC7993146

[17] Fu C Wang M Liang J He T Wang D Xu J. Effectiveness of Lanzhou lamb rotavirus vaccine against rotavirus gastroenteritis requiring hospitalization: a matched case-control study. Vaccine. (2007) 25:8756–61. 10.1016/j.vaccine.2007.10.036 18023510

[18] Zhen SS Li Y Wang SM Zhang XJ Hao ZY Chen Y Effectiveness of the live attenuated rotavirus vaccine produced by a domestic manufacturer in China studied using a population-based case-control design. Emerg Microbes Infect. (2015) 4:e64. 10.1038/emi.2015.64 26576341 PMC4631931

[19] Li J Zhang Y Yang Y Liang Z Tian Y Liu B Effectiveness of Lanzhou lamb rotavirus vaccine in preventing gastroenteritis among children younger than 5 years of age. Vaccine. (2019) 37:3611–6. 10.1016/j.vaccine.2019.03.069 31122857

[20] Xia S Du J Su J Liu Y Huang L Yu Q Efficacy, immunogenicity and safety of a trivalent live human-lamb reassortant rotavirus vaccine (LLR3) in healthy Chinese infants: a randomized, double-blind, placebo-controlled trial. Vaccine. (2020) 38:7393–400. 10.1016/j.vaccine.2020.04.038 32451212

[21] Kirkwood CD Ma LF Carey ME Steele AD. The rotavirus vaccine development pipeline. Vaccine. (2019) 37:7328–35. 10.1016/j.vaccine.2017.03.076 28396207 PMC6892263

[22] Vesikari T Matson DO Dennehy P Van Damme P Santosham M Rodriguez Z Safety and efficacy of a pentavalent human-bovine (WC3) reassortant rotavirus vaccine. N Engl J Med. (2006) 354:23–33. 10.1056/NEJMoa052664 16394299

[23] Jiang V Jiang B Tate J Parashar UD Patel MM. Performance of rotavirus vaccines in developed and developing countries. Hum Vaccin. (2010) 6:532–42. 10.4161/hv.6.7.11278 20622508 PMC3322519

[24] Van Dongen JAP Rouers EDM Schuurman R Band C Watkins SM Van Houten MA Rotavirus vaccine safety and effectiveness in infants with high-risk medical conditions. Pediatrics. (2021) 148:e2021051901. 10.1542/peds.2021-051901 34814164

[25] Ruiz-Palacios GM Pérez-Schael I Velázquez FR Abate H Breuer T Clemens SC Safety and efficacy of an attenuated vaccine against severe rotavirus gastroenteritis. N Engl J Med. (2006) 354:11–22. 10.1056/NEJMoa052434 16394298

[26] Wu ZW Li QL Zhou HS Duan K Gao Z Zhang XJ Safety and immunogenicity of a novel oral hexavalent rotavirus vaccine: a phase I clinical trial. Hum Vaccin Immunother. (2021) 17:2311–8. 10.1080/21645515.2020.1861874 33545015 PMC8189138

[27] Zell R. Picornaviridae-the ever-growing virus family. Arch Virol. (2018) 163:299–317. 10.1007/s00705-017-3614-8 29058149

[28] Wang X An Z Huo D Jia L Li J Yang Y Enterovirus A71 vaccine effectiveness in preventing enterovirus A71 infection among medically-attended hand, foot, and mouth disease cases, Beijing, China. Hum Vaccin Immunother. (2019) 15:1183–90. 10.1080/21645515.2019.1581539 30779680 PMC6605830

[29] Seo D. Estimating the incidence of cases and deaths resulting from hand, foot and mouth disease and its related socioeconomic disease burden in Republic of Korea (2010–2014). Osong Public Health Res Perspect. (2018) 9:112–7. 10.24171/j.phrp.2018.9.3.05 30023155 PMC6037392

[30] Hoang MTV Anh NT Hong NTT Hang VTT Doorn HRV. Clinical and aetiological study of hand, foot and mouth disease in southern Vietnam, 2013–2015: inpatients and outpatients. Int J Infect Dis. (2019) 80:1–9. 10.1016/j.ijid.2018.12.004 30550944 PMC6403263

[31] Li R Liu L Mo Z Wang X Xia J Liang Z An inactivated enterovirus 71 vaccine in healthy children. N Engl J Med. (2014) 370:829–37. 10.1056/NEJMoa1303224 24571755

[32] Guan X Che Y Wei S Li S Zhao Z Tong Y Effectiveness and safety of an inactivated enterovirus 71 vaccine in children aged 6–71 months in a phase IV study. Clin Infect Dis. (2020) 71:2421–7. 10.1093/cid/ciz1114 31734699

[33] Zhu FC Meng FY Li JX Li XL Mao QY Tao H Efficacy, safety, and immunology of an inactivated alum-adjuvant enterovirus 71 vaccine in children in China: a multicentre, randomised, double-blind, placebo-controlled, phase 3 trial. Lancet. (2013) 381:2024–32. 10.1016/S0140-6736(13)61049-1 23726161

[34] Zhu F Xu W Xia J Liang Z Liu Y Zhang X Efficacy, safety, and immunogenicity of an enterovirus 71 vaccine in China. N Engl J Med. (2014) 370:818–28. 10.1056/NEJMoa1304923 24571754

[35] Zhang L Gao F Zeng G Yang H Zhu T Yang S Immunogenicity and safety of inactivated enterovirus 71 vaccine in children aged 36–71 months: a double-blind, randomized, controlled, non-inferiority phase III trial. J Pediatric Infect Dis Soc. (2021) 10:440–7. 10.1093/jpids/piaa129 33269798

[36] Syrogiannopoulos GA Grivea IN Moriondo M Nieddu F Michoula AN Calabrese MR Molecular surveillance of pneumococcal carriage following completion of immunization with the 13-valent pneumococcal conjugate vaccine administered in a 3+1 schedule. Sci Rep. (2021) 11:24534. 10.1038/s41598-021-03720-y 34969968 PMC8718523

[37] Zimmerman RK. Pneumococcal conjugate vaccine for young children. Am Fam Physician. (2001) 63:1991–8. 11388715

[38] Geno KA Saad JS Nahm MH. Discovery of novel pneumococcal serotype 35D, a natural WciG-deficient variant of serotype 35B. J Clin Microbiol. (2017) 55:1416–25. 10.1128/JCM.00054-17 28202800 PMC5405259

[39] Lozano C Torres C. Update on antibiotic resistance in gram-positive bacteria. Enferm Infecc Microbiol Clin. (2017) 35(Suppl 1):2–8. 10.1016/S0213-005X(17)30028-9 28129816

[40] Kim SH Chung DR Song JH Baek JY Thamlikitkul V Wang H Changes in serotype distribution and antimicrobial resistance of Streptococcus pneumoniae isolates from adult patients in Asia: emergence of drug-resistant non-vaccine serotypes. Vaccine. (2020) 38:6065–73. 10.1016/j.vaccine.2019.09.065 31590932

[41] Bröker M Costantino P DeTora L McIntosh ED Rappuoli R. Biochemical and biological characteristics of cross-reacting material 197 CRM197, a non-toxic mutant of diphtheria toxin: use as a conjugation protein in vaccines and other potential clinical applications. Biologicals. (2011) 39:195–204. 10.1016/j.biologicals.2011.05.004 21715186

[42] Romaniuk SI Kolibo DB Komisarenko SV. Perspectives of application of recombinant diphtheria toxin derivatives. Bioorg Khim. (2012) 38:639–52. 10.1134/s106816201206012x 23547467

[43] Southern J Andrews N Sandu P Sheppard CL Waight PA Fry NK Pneumococcal carriage in children and their household contacts six years after introduction of the 13-valent pneumococcal conjugate vaccine in England. PLoS One. (2018) 13:e0195799. 10.1371/journal.pone.0195799 29799839 PMC5969732

[44] Perdrizet J Horn EK Hayford K Grant L Barry R Huang L Historical population-level impact of infant 13-valent pneumococcal conjugate vaccine (PCV13) national immunization programs on invasive pneumococcal disease in Australia, Canada, England and Wales, Israel, and the United States. Infect Dis Ther. (2023) 12:1351–64. 10.1007/s40121-023-00798-x 37079175 PMC10229489

[45] Bruce MG Singleton R Bulkow L Rudolph K Zulz T Gounder P Impact of the 13-valent pneumococcal conjugate vaccine (pcv13) on invasive pneumococcal disease and carriage in Alaska. Vaccine. (2015) 33:4813–9. 10.1016/j.vaccine.2015.07.080 26247901

[46] Lee H Choi EH Lee HJ. Efficacy and effectiveness of extended-valency pneumococcal conjugate vaccines. Korean J Pediatr. (2014) 57:55–66. 10.3345/kjp.2014.57.2.55 24678328 PMC3965795

[47] Plosker GL. 13-valent pneumococcal conjugate vaccine: a review of its use in infants, children, and adolescents. Paediatr Drugs. (2013) 15:403–23. 10.1007/s40272-013-0047-z 24030738

[48] Hu R Liu Y Zhang L Kang G Xu B Li M Post-marketing safety surveillance for both CRM197 and TT carrier proteins PCV13 in Jiangsu, China. Front Public Health. (2023) 11:1272562. 10.3389/fpubh.2023.1272562 37908689 PMC10613985

[49] Yu R Xu J Hu T Chen W. The pneumococcal polysaccharide-tetanus toxin native C-fragment conjugate vaccine: the carrier effect and immunogenicity. Mediators Inflamm. (2020) 2020:9596129. 10.1155/2020/9596129 32714092 PMC7355367

[50] Zhao Y Li G Xia S Ye Q Yuan L Li H Immunogenicity and safety of a novel 13-valent pneumococcal vaccine in healthy Chinese infants and toddlers. Front Microbiol. (2022) 13:870973. 10.3389/fmicb.2022.870973 35615504 PMC9125316

[51] Falugi F Petracca R Mariani M Luzzi E Mancianti S Carinci V Rationally designed strings of promiscuous CD4(+) T cell epitopes provide help to haemophilus influenzae type b oligosaccharide: a model for new conjugate vaccines. Eur J Immunol. (2001) 31:3816–24. 10.1002/1521-4141(200112)31:12<3816::AID-IMMU3816>3.0.CO;2-K 11745403

[52] Baraldo K Mori E Bartoloni A Norelli F Grandi G Rappuoli R Combined conjugate vaccines: enhanced immunogenicity with the N19 polyepitope as a carrier protein. Infect Immun. (2005) 73:5835–41. 10.1128/IAI.73.9.5835-5841.2005 16113302 PMC1231108

[53] Liang Q Li H Chang X Zhang H Hao H Ye Q A phase 3 clinical trial of MINHAI PCV13 in Chinese children aged from 7 months to 5 years old. Vaccine. (2021) 39:6947–55. 10.1016/j.vaccine.2021.09.047 34706841

[54] Bhandari N Rongsen-Chandola T Bavdekar A John J Antony K Taneja S Efficacy of a monovalent human-bovine (116E) rotavirus vaccine in Indian children in the second year of life. Vaccine. (2014) 32(Suppl 1):A110–6. 10.1016/j.vaccine.2014.04.079 25091663

[55] Bhandari N Rongsen-Chandola T Bavdekar A John J Antony K Taneja S Efficacy of a monovalent human-bovine (116E) rotavirus vaccine in Indian infants: a randomised, double-blind, placebo-controlled trial. Lancet. (2014) 383:2136–43. 10.1016/S0140-6736(13)62630-6 24629994 PMC4532697

[56] Kulkarni PS Desai S Tewari T Kawade A Goyal N Garg BS A randomized phase III clinical trial to assess the efficacy of a bovine-human reassortant pentavalent rotavirus vaccine in Indian infants. Vaccine. (2017) 35:6228–37. 10.1016/j.vaccine.2017.09.014 28967523 PMC5651219

[57] Dang DA Nguyen VT Vu DT Nguyen TH Nguyen DM Yuhuan W A dose-escalation safety and immunogenicity study of a new live attenuated human rotavirus vaccine (rotavin-M1) in Vietnamese children. Vaccine. (2012) 30(Suppl 1):A114–21. 10.1016/j.vaccine.2011.07.118 22520120

[58] Anderson EJ Sederdahl BK. Intussusception risk increased after rotavirus vaccination but outweighed by benefits. Evid Based Med. (2014) 19:191–2. 10.1136/eb-2014-101793 24795445

[59] Carlin JB Macartney KK Lee KJ Quinn HE Buttery J Lopert R Intussusception risk and disease prevention associated with rotavirus vaccines in Australia’s national immunization program. Clin Infect Dis. (2013) 57:1427–34. 10.1093/cid/cit520 23964090

[60] Tate JE Yen C Steiner CA Cortese MM Parashar UD. Intussusception rates before and after the Introduction of rotavirus vaccine. Pediatrics. (2016) 138:e20161082. 10.1542/peds.2016-1082 27558938 PMC11973882

[61] Bergman H Henschke N Hungerford D Pitan F Ndwandwe D Cunliffe N. Vaccines for preventing rotavirus diarrhoea: vaccines in use. Cochrane Database Syst Rev. (2021) 11:CD008521. 10.1002/14651858.CD008521.pub6 34788488 PMC8597890

[62] Jiang B Gentsch JR Glass RI. The role of serum antibodies in the protection against rotavirus disease: an overview. Clin Infect Dis. (2002) 34:1351–61. 10.1086/340103 11981731

[63] Groome MJ Fairlie L Morrison J Fix A Koen A Masenya M Safety and immunogenicity of a parenteral trivalent P2-VP8 subunit rotavirus vaccine: a multisite, randomised, double-blind, placebo-controlled trial. Lancet Infect Dis. (2020) 20:851–63. 10.1016/S1473-3099(20)30001-3 32251641 PMC7322558

[64] Resch TK Wang Y Moon SS Joyce J Li S Prausnitz M Inactivated rotavirus vaccine by parenteral administration induces mucosal immunity in mice. Sci Rep. (2018) 8:561. 10.1038/s41598-017-18973-9 29330512 PMC5766576

[65] Estes MK Crawford SE Penaranda ME Petrie BL Burns JW Chan WK Synthesis and immunogenicity of the rotavirus major capsid antigen using a baculovirus expression system. J Virol. (1987) 61:1488–94. 10.1128/jvi.61.5.1488-1494.1987 3033276 PMC254127

[66] Madore HP Estes MK Zarley CD Hu B Parsons S Digravio D Biochemical and immunologic comparison of virus-like particles for a rotavirus subunit vaccine. Vaccine. (1999) 17:2461–71. 10.1016/S0264-410X(98)00319-3 10392629

[67] Blazevic V Malm M Arinobu D Lappalainen S Vesikari T. Rotavirus capsid VP6 protein acts as an adjuvant in vivo for norovirus virus-like particles in a combination vaccine. Hum Vaccin Immunother. (2016) 12:740–8. 10.1080/21645515.2015.1099772 26467630 PMC4964741

[68] Noad R Roy P. Virus-like particles as immunogens. Trends Microbiol. (2003) 11:438–44. 10.1016/S0966-842X(03)00208-7 13678860

[69] Mao Q Wang Y Bian L Xu M Liang Z. EV-A71 vaccine licensure: a first step for multivalent enterovirus vaccine to control HFMD and other severe diseases. Emerg Microbes Infect. (2016) 5:e75. 10.1038/emi.2016.73 27436364 PMC5141264

[70] He F Rui J Deng Z Zhang Y Qian K Zhu C Surveillance, epidemiology and impact of EV-A71 vaccination on hand, foot, and mouth disease in Nanchang, China, 2010–2019. Front Microbiol. (2021) 12:811553. 10.3389/fmicb.2021.811553 35069515 PMC8770912

[71] Xie J Yang XH Hu SQ Zhan WL Zhang CB Liu H. Co-circulation of coxsackieviruses A-6, A-10, and A-16 causes hand, foot, and mouth disease in Guangzhou city, China. BMC Infect Dis. (2020) 20:271. 10.1186/s12879-020-04992-x 32264839 PMC7137261

[72] Ji T Guo Y Huang W Shi Y Xu Y Tong W The emerging sub-genotype C2 of coxsackievirusA10 associated with hand, foot and mouth disease extensively circulating in mainland of China. Sci Rep. (2018) 8:13357. 10.1038/s41598-018-31616-x 30190558 PMC6127217

[73] Yang Q Ding J Cao J Huang Q Hong C Yang B. Epidemiological and etiological characteristics of hand, foot, and mouth disease in Wuhan, China from 2012 to 2013: outbreaks of coxsackieviruses A10. J Med Virol. (2015) 87:954–60. 10.1002/jmv.24151 25754274

[74] Guan H Wang J Wang C Yang M Liu L Yang G Etiology of multiple non-EV71 and non-CVA16 enteroviruses associated with hand, foot and mouth disease in Jinan, China, 2009–June 2013. PLoS One. (2015) 10:e0142733. 10.1371/journal.pone.0142733 26562154 PMC4642993

[75] Huang Y Zhou Y Lu H Yang H Feng Q Dai Y Characterization of severe hand, foot, and mouth disease in Shenzhen, China, 2009–2013. J Med Virol. (2015) 87:1471–9. 10.1002/jmv.24200 25951788

[76] Duan X Zhang C Wang X Ren X Peng H Tang X Molecular epidemiology and clinical features of hand, foot and mouth disease requiring hospitalization after the use of enterovirus A71 inactivated vaccine in Chengdu, China, 2017–2022: a descriptive study. Emerg Microbes Infect. (2022) 11:2510–9. 10.1080/22221751.2022.2125346 36103331 PMC9621254

[77] Lim H In HJ Lee JA Sik Yoo J Lee SW Chung GT The immunogenicity and protection effect of an inactivated coxsackievirus A6, A10, and A16 vaccine against hand, foot, and mouth disease. Vaccine. (2018) 36:3445–52. 10.1016/j.vaccine.2018.05.005 29739716

[78] Zhang Z Dong Z Wang Q Carr MJ Li J Liu T Characterization of an inactivated whole-virus bivalent vaccine that induces balanced protective immunity against coxsackievirus A6 and A10 in mice. Vaccine. (2018) 36:7095–104. 10.1016/j.vaccine.2018.09.069 30316529

[79] Caine EA Fuchs J Das SC Partidos CD Osorio JE. Efficacy of a trivalent hand, foot, and mouth disease vaccine against enterovirus 71 and coxsackieviruses A16 and A6 in mice. Viruses. (2015) 7:5919–32. 10.3390/v7112916 26593938 PMC4664989

[80] Chackerian B. Virus-like particles: flexible platforms for vaccine development. Expert Rev Vaccines. (2007) 6:381–90. 10.1586/14760584.6.3.381 17542753

[81] Dai W Xiong P Zhang X Liu Z Chen J Zhou Y Recombinant virus-like particle presenting a newly identified coxsackievirus A10 neutralization epitope induces protective immunity in mice. Antiviral Res. (2019) 164:139–46. 10.1016/j.antiviral.2019.02.016 30817941

[82] Chen X Zhang Y Mao N Zhu S Ji T Xu W. Intranasal immunization with coxsackievirus A16 virus-like particles confers protection against lethal infection in neonatal mice. Arch Virol. (2019) 164:2975–84. 10.1007/s00705-019-04418-3 31570994

[83] Wang Z Zhou C Gao F Zhu Q Jiang Y Ma X Preclinical evaluation of recombinant HFMD vaccine based on enterovirus 71 (EV71) virus-like particles (VLP): immunogenicity, efficacy and toxicology. Vaccine. (2021) 39:4296–305. 10.1016/j.vaccine.2021.06.031 34167837

[84] Ku Z Liu Q Ye X Cai Y Wang X Shi J A virus-like particle based bivalent vaccine confers dual protection against enterovirus 71 and coxsackievirus A16 infections in mice. Vaccine. (2014) 32:4296–303. 10.1016/j.vaccine.2014.06.025 24950363

[85] Lai X Wahl B Yu W Xu T Zhang H Garcia C National, regional, and provincial disease burden attributed to Streptococcus pneumoniae and haemophilus influenzae type b in children in China: modelled estimates for 2010–17. Lancet Reg Health West Pac. (2022) 22:100430. 10.1016/j.lanwpc.2022.100430 35308577 PMC8928075

[86] Ebell MH. Community-Acquired pneumonia: determining safe treatment in the outpatient setting. Am Fam Physician. (2019) 99:768–9. 31194484

[87] Pomat WS van den Biggelaar AHJ Wana S Francis JP Solomon V Greenhill AR Safety and immunogenicity of pneumococcal conjugate vaccines in a high-risk population: a randomized controlled trial of 10-valent and 13-valent pneumococcal conjugate vaccine in Papua New Guinean infants. Clin Infect Dis. (2019) 68:1472–81. 10.1093/cid/ciy743 30184183 PMC6481999

[88] Gruber WC Scott DA Emini EA. Development and clinical evaluation of prevnar 13, a 13-valent pneumocococcal CRM197 conjugate vaccine. Ann N Y Acad Sci. (2012) 1263:15–26. 10.1111/j.1749-6632.2012.06673.x 22830997

[89] Wang J Bai S Zhou S Zhao W Li Q Lv M Immunogenicity and safety of 7-valent pneumococcal conjugate vaccine (PCV7) in children aged 2–5 years in China. Vaccine. (2021) 39:3428–34. 10.1016/j.vaccine.2021.04.037 33965257

[90] Sartori AM de Soárez PC Novaes HM. Cost-effectiveness of introducing the 10-valent pneumococcal conjugate vaccine into the universal immunisation of infants in Brazil. J Epidemiol Community Health. (2012) 66:210–7. 10.1136/jech.2010.111880 20884668

[91] Bricks LF Berezin E. Impact of pneumococcal conjugate vaccine on the prevention of invasive pneumococcal diseases. J Pediatr. (2006) 82:S67–74. 10.2223/JPED.1475 16721440

[92] Kim YK LaFon D Nahm MH. Indirect effects of pneumococcal conjugate vaccines in national immunization programs for children on adult pneumococcal disease. Infect Chemother. (2016) 48:257–66. 10.3947/ic.2016.48.4.257 28032483 PMC5204004

[93] Chapman TJ Olarte L Dbaibo G Houston AM Tamms G Lupinacci R PCV15, a pneumococcal conjugate vaccine, for the prevention of invasive pneumococcal disease in infants and children. Expert Rev Vaccines. (2024) 23:137–47. 10.1080/14760584.2023.2294153 38111990

[94] Ishihara Y Fukazawa M Enomoto S de Solom R Yamaji M Kline M A phase 3 randomized study to evaluate safety and immunogenicity of 20-valent pneumococcal conjugate vaccine in healthy Japanese infants. Int J Infect Dis. (2024) 141:106942. 10.1016/j.ijid.2024.01.009 38242195

[95] Senders S Klein NP Tamimi N Thompson A Baugher G Trammel J A phase three study of the safety and immunogenicity of a four-dose series of 20-valent pneumococcal conjugate vaccine in healthy infants. Pediatr Infect Dis J. (2024) 43:596–603. 10.1097/INF.0000000000004334 38535409 PMC11090512

[96] Alderson MR. Status of research and development of pediatric vaccines for *Streptococcus pneumoniae*. Vaccine. (2016) 34:2959–61. 10.1016/j.vaccine.2016.03.107 27083428 PMC4906266

[97] Dagan R. Relationship between immune response to pneumococcal conjugate vaccines in infants and indirect protection after vaccine implementation. Expert Rev Vaccines. (2019) 18:641–61. 10.1080/14760584.2019.1627207 31230486

[98] Lafnitzegger A Gaviria-Agudelo C. Vaccine hesitancy in pediatrics. Adv Pediatr. (2022) 69:163–76. 10.1016/j.yapd.2022.03.011 35985708

